# Neurobiological activity of the auditory pathway and memory: a comparative study in individuals infected with SARS-Cov-2

**DOI:** 10.1590/2317-1782/e20250056en

**Published:** 2026-03-27

**Authors:** Liliane Razador Kerkhoff, Hélinton Goulart Moreira, Larissa Coradini, Fabiana Cristina Toillier, Cristiane Laís Piccoloto, Denis Altieri de Oliveira Moraes, Michele Vargas Garcia

**Affiliations:** 1 Programa de Pós-graduação em Distúrbios da Comunicação Humana, Departamento de Fonoaudiologia, Universidade Federal de Santa Maria – UFSM - Santa Maria (RS), Brasil.; 2 Curso de Fonoaudiologia, Universidade Federal de Santa Maria – UFSM - Santa Maria (RS), Brasil.

**Keywords:** SARS-Cov-2, Cognition, Adults, Electrophysiology, Memory

## Abstract

**Purpose:**

To analyze the influence of SARS-CoV-2 on memory and on the components of the Long Latency Auditory Evoked Potential.

**Methods:**

Quantitative descriptive study, approved by the Research Ethics Committee under the number 56038322100005346. For the sample composition, 32 participants were invited, aged between 18 and 35 years, without hearing loss, middle ear integrity and contralateral acoustic reflexes present bilaterally, were invited to compose the sample. In addition, they were free from neurological or psychiatric alterations, with or without a diagnosis of SARS-CoV-2. Thirty-two individuals participated in the study, 24 (75%) females and eight (25%) males, distributed in two groups. The Study Group (EG) consisted of 15 subjects (53%) diagnosed with SARS-CoV-2 and the Control Group (CG) consisted of 17 subjects (47%) without such diagnosis. All participants underwent anamnesis, meatoscopy, basic audiological evaluation, Long Latency Auditory Evoked Potential (LLAEP) and the Brief Neuropsychological Assessment Instrument-NEUPSILIN, using the total score for each memory subtype. Finally, for statistical analysis of the data, the Student's T-test was used, adopting a p-value <0.05.

**Results:**

It was possible to observe significant differences with lower performance for the study group in Episodic-Semantic Visual Memory (p-value = 0.014) and for Short-Term Visual Memory (p-value = 0.042). In the LLAEP there was a significant difference only for the P1 component, with lower latency in the EG.

**Conclusion:**

SARS-CoV-2 infection negatively influenced the memory of young adults, mainly in episodic-semantic verbal memory and short-term visual memory. Furthermore, the LLAEP contributed to the diagnosis of these participants by demonstrating the deleterious effects of the infection.

## INTRODUCTION

Since December 2019, the world has been experiencing a worrying situation in terms of public health due to the emergence of a highly transmissible virus, which has led to a surge in cases worldwide. In February 2020, the World Health Organization (WHO)^(1)^ officially named the disease COVID-19 (*Coronavirus Disease 2019*), as well as demonstrated the various consequences of such respiratory infection for global human health and in March of the same year declared a pandemic.

Based on the pathophysiology of the viral infection and its various manifestations, even after the acute phase, in 2021, the WHO named the various sequelae of the SARS-CoV-2 virus as Post-COVID-19 Syndrome, Long COVID, or Post-acute Sequelae of COVID-19, which define people who still have symptoms after infection with the virus^(2-4)^.

Given this, we know of the various impacts that SARS-CoV-2 can cause, such as problems related to hearing acuity, otological symptoms such as dizziness and tinnitus, difficulties in understanding speech, and even memory impairment, which is often reported in speech therapy clinics. Thus, these impacts may be related to structural changes that manifest themselves in different functional capacities of individuals. Through the medulla, the SARS-CoV-2 virus, via neurotropism, is able to infect and spread the virus to the central nervous system (CNS), infecting cortical and hypothalamic regions^(5)^. Since the hypothalamus connects to most brain regions, impairments in attention, executive functions, and memory may be manifested in the individual^(6)^. Furthermore, memory loss can be explained by the term *"brain fog*," which refers to a set of neurological symptoms related to COVID-19 that causes short- and long-term memory loss in many patients^(2)^.

To evaluate the Central Auditory Nervous System (CANS), we have the Long Latency Auditory Evoked Potential (LLAEP). This evaluates auditory function in terms of synaptic processes at the cortical and cognitive levels, investigating skills such as attention and memory. The potentials obtained through LLAEP are captured objectively and non-invasively. The P1, N1, and P2 components reflect the arrival of the stimulus- t the cortex, while the N2 component is related to the abilities of perception, discrimination, recognition, and classification of the sound stimulus. Finally, P3 (or P300) makes it possible to understand whether the individual focused on performing the proposed activity, reflecting on attention, discrimination, integration, and memory skills^(7)^.

To assess cognitive functionality, the brief neuropsychological test NEUPSILIN^(8)^ aims to evaluate multiple cognitive abilities.

In the literature, there are still no studies that have verified the findings of NEUPSILIN with LLAEP as the most effective assessment method for diagnosing memory complaints in patients after SARS-CoV-2 infection. One study^(9)^ used the brief neuropsychological assessment NEUPSILIN and Cognitive Potential - P300 with speech stimulation, pre- and post-cognitive auditory training, to verify whether its therapeutic strategy was effective in treating adults with complaints of speech comprehension and cognition after COVID-19 infection.

Thus, this study is justified by the need to identify which diagnostic assessment technique (behavioral and electrophysiological) is most effective in detecting changes in a population of young adults with post-COVID-related complaints.

Therefore, the objective of the present study was to analyze the influence of SARS-CoV-2 on different memory subtypes and Long Latency Auditory Evoked Potential (LLAEP-verbal) in young adults.

## METHOD

### Study design

This is a descriptive and quantitative study, approved by the Research Ethics Committee under number 56038322100005346. The Free and Informed Consent Form (FICF) was signed by all study participants, aiming to clarify the risks and benefits of their participation.

The following eligibility criteria were listed: individuals of both sexes, aged between 18 and 35 years, educated, speakers of Brazilian Portuguese, right-handed, hearing thresholds within normal standards, middle ear integrity and contralateral acoustic reflexes present bilaterally, with or without a diagnosis of COVID-19, confirmed by RT-PCR testing.

Individuals with evident or diagnosed neurological or psychiatric impairment, musicians, individuals exposed to noise, or with otological complaints (dizziness and tinnitus) were excluded.

### Participants

The sample was selected for convenience, with 85 individuals attending the audiology outpatient clinic of a teaching clinic between September 2021 and March 2022. Of these, 47 participants were excluded, as 36 reported chronic tinnitus (42.35%), nine had hearing loss (10.58%), five (5.88%) were unable to complete the assessments, and two (2.35%) had middle ear disorders. Thus, there were 32 individuals, 24 females and eight males, matched for age, sex, and education and divided into two groups:

Experimental group (EG), composed of 15 individuals (53%), aged between 19 and 27 years (mean: 22.54), including 12 women and five men, with a minimum of 12 years and a maximum of 18 years of schooling (mean: 15.38 years), diagnosed with SARS-CoV-2 infection (confirmed by RT-PCR testing);

Control Group (CG), composed of 17 participants (47%), including 12 females and three males, aged between 19 and 34 years (mean: 22.79), with a minimum of 14 years and a maximum of 17 years of schooling (average: 15.38 years) and without a diagnosis of SARS-CoV-2 infection (confirmed by RT-PCR testing).

### Methodological design

The procedures were divided into: procedures for sample composition and research procedures. All EG procedures were performed on average six months after infection.

### Procedures for sample composition

Semi-structured medical history: All individuals completed an online questionnaire with their identification data, questions about hearing aspects, audiological complaints of speech comprehension, cognitive aspects, as well as general health.

Visual inspection of the external auditory canal: Performed with a Mikatos TK otoscope to assess the conditions necessary for the subsequent examination, as well as the possible need for referral to an otolaryngologist.

Tonal Threshold Audiometry (ATL): Performed in an acoustically treated booth, with TDH-39 headphones. To test hearing thresholds, the *Interacoustics* AD229 audiometer was used*.* Frequencies from 250Hz to 8000Hz were evaluated by air conduction, taking into account the normality proposed by WHO^(10)^, i.e., with tonal thresholds up to 20 dBHL.

Speech audiometry: Performed using the same equipment as for ATL. The evaluation was performed in two stages, both conducted aloud. First, the Speech Recognition Threshold (SRT) was performed, initially at an intensity of 30 dBHL above the individual's tritonal average. The descending-ascending technique was used, considering as the threshold the point at which the individual correctly identified 50% of the four presentations. Next, the Speech Recognition Percentage Index (SRPI) was applied, setting the intensity at 40 dBHL above the tritonal average, considering the participant's comfort. For this, 25 words were presented to the participant, who had to repeat them, with each error equivalent to 4%. The errors were subtracted from the total of 100%.

Acoustic Imitance Measurements (AIM): These were performed using *Interacoustics* AT235 equipment and TDH-39 headphones. For the analysis of tympanometric curves, the classification criteria of Jerger, Jerger, and Mauldin^(11)^ were used, while acoustic reflexes were classified based on the criteria of Jerger and Jerger^(12)^ .

### Research procedures

Brief Neuropsychological Assessment Instrument (NEUPSILIN): This was used to assess cognitive memory ability, as it is a clinical tool that allows for a comprehensive description of the main components of cognitive abilities across different age groups. It can be applied to individuals aged 12 to 90 (from adolescence to old age), with at least one year of school . The test consists of 32 tasks that aim to characterize a brief cognitive profile based on eight main cognitive functions: temporal-spatial orientation, concentrated auditory attention, visual perception, memory, arithmetic skills, language, praxis, and executive functions.

The assessment protocol consists of the application manual, the stimulus book, along with specific materials for each task. During the test, the evaluator can repeat the instructions for each subtest according to the individual's needs, except for attention and memory tasks. Since the focus of this study was to analyze memory ability, only this subtest was used as data. The instrument was administered individually, with one evaluator dedicated to each participant, and lasted approximately 40 minutes in a quiet environment. The reference values used were proposed in the study by Fonseca et al.^(8)^ .

In the memory assessment, the instrument evaluates the following types: working memory (ascending order of digits and auditory span of words in sentences), episodic verbal memory (immediate, delayed, and recognition), semantic memory (long-term), visual memory (short-term), and prospective memory. In each of the activities, the individual was instructed according to the instruction book. According to the general rules of the protocol, in the subtests evaluated in this study, a single self-correction was accepted for all tasks and items.

The final score was obtained through the complete score for total memory ability. For memory subtypes, total scores were used, through the sum of each subtask within the types of memories evaluated, with the higher the score, the better the participant's cognitive performance.

Long Latency Auditory Potential-verbal (LLAEP): The LLAEP-verbal was applied to compare the two groups, with the aim of obtaining information about the neural functioning of the CANS, through the analysis of amplitudes (in *microvolts*) and the P2/P1 wave ratio. The equipment used was the Smart EP from *Intelligent Hearing System,* and the electrodes were positioned at points Fpz, A1, A2, and Cz.

One hundred and fifty verbal stimuli (*sweeps*) were presented at an intensity of 70 dBHL, composed of the syllable /ba/, which represented the frequent stimulus (80% of the time), and the syllable /di/, which represented the rare stimulus (20% of the time), in the traditional *oddball* paradigm. The stimulus speed was 1.1/sec, with a 1-30 Hz filter, 100K gain, and a 510 ms time window. The subjects remained alert and paid attention to the "rare" stimuli, as well as counting these stimuli mentally.

The ears were tested binaurally, triggering responses from the right and left ears. From the stimulation, two waves (frequent wave and rare wave) were generated, and the latency and amplitude of the potentials in each ear were analyzed for subsequent comparisons. The cortical potentials (P1, N1, P2) and the mixed potential (N2) were marked on the frequent trace, and the cognitive potential (P300) was marked on the rare trace. The reference values used were proposed in the study by Bruno et al.^(13)^ and Didoné et al.^(14)^ .

### Statistical analysis of data

According to the final size of the groups, the power obtained by the test for comparing means between two independent groups was computed using the G*Power program. The parameters used were a large effect size (0.8), a significance level of 5%, and sample sizes set at 15 and 17 individuals. Thus, the final estimated power of the test is approximately 71%, which is considered a satisfactory level of accuracy. The data were tabulated in an *Excel* spreadsheet and imported for statistical and quantitative analysis in the SPSS v. 22 program^(15)^. The assumption of normality of the variables was verified by the Kolmogorov-Smirnov test, and this hypothesis was not rejected for any variable in the study (p > 0.05). Thus, the T-test for independent samples was used, considering p ≤ 0.05 as the statistical significance value.

## RESULTS

In Table 1, to observe the homogeneity of the sample, comparisons were made between the variables between the groups, showing no statistically significant differences.

**Table 1:** Analysis of age and education variables between both groups

**VARIABLES**	**GROUP**	**N**	**MIN**	**MAX**	**AVERAGE**	**SD**	**P-VALUE**
AGE	CG	17	19.0	34.0	22.5	3.9	0.997
	EG	15	19.0	27.0	22.5	2.2	
EDUCATION	CG	17	14.0	17	14.9	1.1	0.213
	EG	15	12.0	18.0	15.5	1.5	

**Caption:** CG = Control Group; EG = Experimental Group; MIN = Minimum; MAX = Maximum; SD = Standard Deviation; N = Number of participants

In Table 2, comparisons were made between the total score and memory subtypes, showing significant differences between episodic-semantic verbal memory and short-term visual memory, with lower scores in the group with SARS-CoV-2 infection.

**Table 2:** Analysis of the different memory subtests in Neupsilin

**VARIABLES**	**GROUP**	**N**	**MIN**	**MAX**	**AVERAGE**	**SD**	**P-VALUE**
Working Memory	CG	17	20.0	36.0	28.6	4.1	0.341
	EG	15	21.0	36.0	30.1	4.4	
Verbal Episodic-Semantic Memory	CG	17	16.0	35	24.8	6.7	**0.063**
	EG	15	16.0	28.8	21.0	3.4	
Long-Term Semantic Memory	CG	17	4.0	5.0	4.8	0.4	0.260
	EG	15	4.0	5.0	4.9	0.2	
Short-Term Visual Memory	CG	17	3.0	3	3.0	-	**0.041** ^*^
	EG	15	2.0	3.0	2.8	0.4	
Prospective Memory	CG	17	1.0	2.0	1.9	0.4	0.487
	EG	15	1.0	2.0	1.9	0.2	
Total Memory	CG	17	45.0	79.0	63.1	10	0.374
	EG	15	47.0	72.0	60.4	6.9	

* Significant difference

**Caption:** CG = Control Group; EG = Experimental Group; MIN = minimum; MAX = maximum; SD = standard deviation; N = number of individuals

In Table 3, the means between the groups were compared for the LLAEP-verbal components, showing a significant difference between the groups for latency and amplitude of the P1 component. The difference between the number of ears is noteworthy, as some research participants had no potential. Even though the stimulation was binaural, responses were obtained from both ears. However, the table shows that the right and left ears were agglutinated, as there was no significant difference between them in the comparison of latency and amplitude of the components between the ears.

**Table 3:** Analysis of LLAEP-verbal between groups

**VARIABLES**	**GROUP**	**N**	**MIN**	**MAX**	**AVERAGE**	**SD**	**P-VALUE**
P1 - L	CG	33	50.0	95.0	71.6	12.6	**0.034**
	EG	29	36.0	110.0	59.9	16.7	
P1 - A	CG	33	0.5	5.8	2.9	1.6	**0.004** ^*^
	EG	29	1.1	6.9	4.5	1.3	
N1 - L	CG	34	90.0	148.0	110.5	10.6	0.745
	EG	30	86.0	149.0	108.9	15.8	
N1 - A	CG	34	2.1	8.5	5.3	2.0	0.290
	EG	30	2.8	11.5	6.2	2.2	
P2 - L	CG	34	155.0	202.0	179.2	12.7	0.374
	EG	30	149.0	224.0	174.1	18.2	
P2 - A	CG	34	0.9	13.2	6.0	3.4	0.396
	EG	30	1.0	10.0	5.2	2.5	
N2 - L	CG	33	217.0	318.0	255.0	24.2	0.504
	EG	30	205.0	296.0	248.8	25.8	
N2 - A	CG	33	0.2	8.7	3.5	2.9	0.991
	EG	30	0.4	9.1	3.5	2.4	
P3 - L	CG	34	226.0	359.0	297.9	37.6	0.134
	EG	24	226.0	384.0	318.8	36.5	
P3 - A	CG	34	1.9	10.8	5.6	2.7	0.625
	EG	24	1.1	16.0	5.0	4	

* Significant difference

**Caption:** L = latency; A = amplitude; CG = control group; EG = experimental group; MIN = minimum; MAX = maximum; SD = standard deviation; N = number of ears

**Figure 1:**
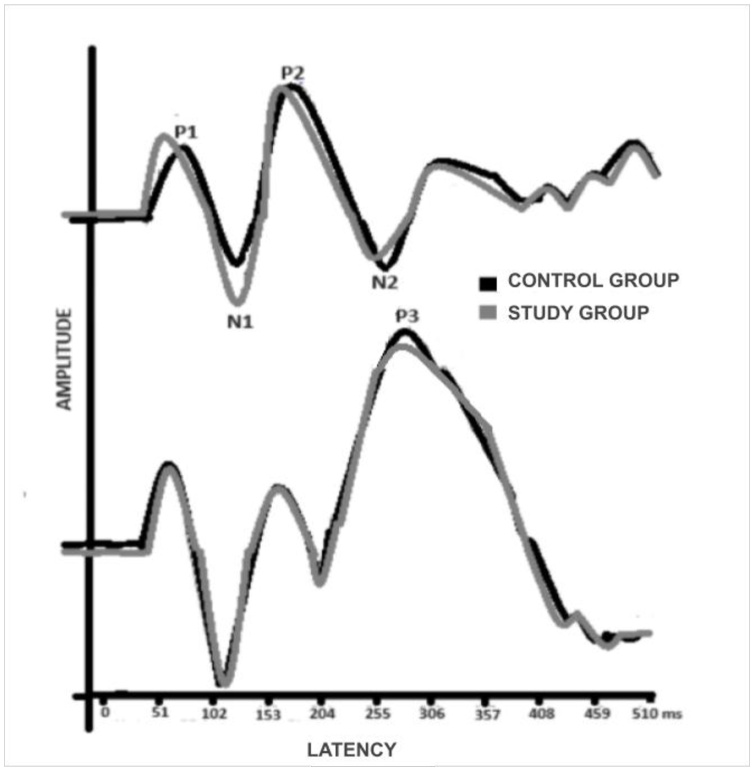
Representation of LLAEP-verbal for both groups

Source: Image from the researcher's personal collection

Figure 1 shows the representation of the verbal LLAEP for both groups.

Table 4 shows the analysis of the presence and absence of components by group and total sum of the ears, demonstrating a higher occurrence of absences for P3 in relation to the EG.

**Table 4:** Analysis of relative frequency of occurrences of presence and absence of LLAEP components between groups

**LLAEP-(ms)**	**Occurrence**	**EG (30 ears) N %**	**CG (34 ears) N %**
**P1**	Absence	1 3.34%	1 2.94%
	Presence	29 96.66%	33 97.06%
**N1**	Absence	0 0%	0 0%
	Presence	30 100%	34 100%
**P2**	Absence	0 0%	0 0%
	Presence	30 100%	34 100%
**N2**	Absence	0 0%	1 2.94%
	Presence	30 100%	33 97.06%
**P3**	Absence	6 20%	0 0%
	Presence	24 80%	34 100%

**Caption:** EG = Experimental Group; CG = Control Group

## DISCUSSION

The relevance of this study refers to the fact that numerous individuals in the post-infection period of SARS-CoV-2 present with memory complaints, which is a persistent symptom in 70% of the participants analyzed^(16)^ . Thus, after evaluating this complaint in detail, the data observed are consistent with the hypothesis of this study that individuals diagnosed with SARS-CoV-2 have a greater number of memory changes.

Table 1 shows that the participants in this study are all young and highly educated, which makes it even more relevant to assess memory and neuronal functioning of the central nervous system (CNS) after SARS-CoV-2 infection. In this context, understanding the functioning of the CNS in this population can help identify possible discrepancies in information processing or early signs of neuropsychological changes, contributing to the prevention of cognitive disorders throughout life.

Table 2 shows worse findings in the different memory subtypes for the group after SARS-CoV-2 infection, with two of the six tasks showing statistical significance, namely episodic-semantic verbal memory and short-term visual memory. This data agrees with scholars who have demonstrated a lag in episodic verbal memory ability in individuals post-SARS-CoV-2 infection when compared to healthy or asymptomatic patients^(17-19)^ . Furthermore, the data presented here are consistent with the study by Bhattacharyya et al.^(20)^ , which reported that individuals post-SARS-CoV-2 infection may experience reduced alertness.

Another study conducted five months after hospital discharge for SARS-CoV-2 demonstrated cognitive abnormalities in individuals, with approximately 20% of the sample presenting verbal memory dysfunction after such infection^(21)^. This occurs because verbal processes require memory, as their basic function is to structure thought in an orderly manner, allowing for the retention, recall, and reproduction of content^(22)^.

With regard to short-term visual memory, there is a relationship with the physiology of explicit memory, which is dependent on mechanisms such as the hippocampus, cerebral amygdala, and prefrontal cortex, mainly. Studies report that there is brain damage caused by SARS-CoV-2, highlighting inflammatory processes and neuron death^(23)^, once again justifying the findings in Table 2.

Regarding the LLAEP-verbal data observed in Table 3, with a difference between the groups in the P1 Potential, we can observe that lower latency and higher amplitude were obtained for the EG. This data shows us greater speed in neural activation (ms) and greater neural recruitment (amplitude) in the primary auditory cortex. Here we observe that the increase in amplitude in relation to the EG group may not be a positive factor. The study by Oliveira et al.^(7)^ found greater amplitudes for P1 in the elderly compared to the adult group, demonstrating greater robustness of neural response to compensate for other possible deficits. They suggested that a greater amplitude of the cortical potential translates into greater auditory effort, which consequently causes greater fatigue leading to mental exhaustion, which may be related to cognitive impairment, correlating with the attention and memory complaints of the individuals studied.

Furthermore, it is known that the P1 component is a biomarker that can be identified in all age groups and is directly related to sound detection in the primary auditory cortex, being the first record of sound signal processing^(24)^ . Although the objective of this study is not the valuation of Central Auditory Processing, the inseparability of auditory and cognitive skills is well known and evident. In another study^(25)^, P1 showed 57.5% accuracy in relation to the probability of alteration in auditory processing, while P3 showed 55%. The data presented here are consistent with the above study in terms of the importance of analyzing all components of the LLAEP and not just P300.

The analysis of P300 provides information on attention, discrimination, integration, and memory, thus demonstrating the functional use that individuals make of auditory stimuli. It is therefore highly related to cognitive skills, especially attention and auditory discrimination^(14)^. Given this, in the context of the present study, the findings obtained in the P3 potential presented in Table 4 are justified.

Also in Table 4, it is possible to observe that there was a 20% absence of P300 in the EG. To ensure that the result was reliable, the evaluator asked the patient about the number of stimuli heard at the end. This finding corroborates the complaint presented by the group participants, highlighting the importance of P300 assessment. This potential is an effective tool for monitoring changes in the Central Auditory System (CAS), with its latency delay or absence being indicative of a disorder related to attention and cognition^(26)^.

The limitations of this study include the fact that no behavioral assessment of Central Auditory Processing was performed, so that individuals with alterations and interest in rehabilitation could be referred for auditory training. A correlation analysis between these behavioral and electrophysiological findings by individuals has not yet been performed, as was done in the study by Oliveira et al.^(7)^ .

Thus, this research emphasizes the importance of including behavioral tests and electrophysiological exams in the diagnosis, since NEUPSILIN and LLAEP-verbal aided in this assessment, allowing for a more accurate intervention. In this sense, these findings agree with Soares et al.^(9)^ , who presented a cognitive auditory training model with visual, auditory, and executive function tasks for the adult population after exposure to SARS-CoV-2, achieving success through integrated rehabilitation (cognition + auditory skills).

## CONCLUSION

SARS-CoV-2 infection negatively influenced the memory of young adults, especially episodic-semantic verbal memory and short-term visual memory. Furthermore, the LLAEP-verbal contributes to the diagnosis of these individuals by demonstrating the deleterious effects of the infection.

## References

[1] OMS: Organização Mundial da Saúde (1948). Constituição..

[2] Fernandes ACM, Alpes MF, Santos CM (2024). Síndrome Pós-COVID-19: investigação de sintomas fonoaudiológicos. Rev CEFAC.

[3] Greenhalgh T, Knight M, A’Court C, Buxton M, Husain L (2020). Management of post-acute covid-19 in primary care. BMJ.

[4] Munipalli B, Seim L, Dawson NL, Knight D, Dabrh AMA (2022). Post-acute sequelae of COVID-19 (PASC): a meta-narrative review of pathophysiology, prevalence, and management. SN Compr Clin Med.

[5] Lima IN, Yamamoto CY, Luz JS, Souza TC, Pereira KF (2022). Perda de memória associada à infecção viral por SARS-CoV-2: revisão de literatura. Res.Soc. Develop..

[6] Nakamura A, Farrer TJ, Liu A (2022). Sequelas a longo prazo em pacientes jovens convalescentes com COVID-19: relatos de casos em medicina neurológica. Case Rep Neurol Med.

[7] Oliveira MFF, Menezes PL, Carnaúba ATL, Pereira LD, Andrade KCL, Frizzo ACF (2021). Cognitive performance and long-latency auditory evoked potentials: a study on aging. Clinics.

[8] Fonseca RP, Salles JF, Parente MAP (2009). Instrumento de Avaliação Neuropsicológica Breve NEUPSILIN..

[9] Soares LS, Malavolta VC, Sanfins MD, Sleifer P, Didoné DD, Garcia MV (2023). Treinamento auditivo cognitivo em sujeitos após COVID-19: uma análise dos efeitos da intervenção em adultos. Audiol Commun Res.

[10] OMS: Organização Mundial de Saúde (2021). Guia de orientação na avaliação audiológica..

[11] Jerger J, Jerger S, Mauldin L (1972). Studies in impedance audiometry: normal and sensorineural ears. Arch Otolaryngol.

[12] Jerger S, Jerger J (1989). Alterações auditivas: um manual para avaliação clínica..

[13] Bruno RS, Oppitz SJ, Garcia MV, Biaggio EPV (2016). Potencial evocado auditivo de longa latência: diferenças na forma de contagem do estímulo raro. Rev CEFAC.

[14] Didoné DD, Garcia MV, Oppitz SJ, Silva TFF, Santos SN, Bruno RS (2016). Potencial evocado auditivo P300 em adultos: valores de referência. Einstein.

[15] IBM Corporation (2013). IBM SPSS statistics for Windows, version 22.0..

[16] Dias BC, Evangelista LCR, Mazer GHC, Ferraz ACPF, Tahan VC, Higa EFR (2022). Alterações cognitivas e de memória na COVID-19: Revisão Integrativa da Literatura. New Trends Qual Res.

[17] Baig AM, Khaleeq A, Ali U, Syeda H (2020). Evidence of the COVID-19 virus targeting the CNS: tissue distribution, host-virus interaction, and proposed neurotropic mechanisms. ACS Chem Neurosci.

[18] Payus AO, Liew Sat Lin C, Mohd Noh M, Jeffree MS, Ali RA (2020). SARS-CoV-2 infection of the nervous system: A review of the literature on neurological involvement in novel coronavirus disease-(COVID-19). Bosn J Basic Med Sci.

[19] Widmann CN, Wieberneit M, Bieler L, Bernsen S, Gräfenkämper R, Brosseron F (2021). Longitudinal neurocognitive and pulmonological profile of long COVID-19: protocol for the COVIMMUNE-clin study. JMIR Res Protoc.

[20] Bhattacharyya R, Upadhya SS, Prabhu P (2024). Effect of COVID-19 on peripheral and central hearing abilities. Egypt J Otolaryngol.

[21] Ferrucci R, Dini M, Groppo E, Rosci C, Reitano MR, Bai F (2021). Long-lasting cognitive abnormalities after COVID-19. Brain Sci.

[22] Acencio CF, Shimazaki EM (2022). O processo da linguagem escrita de alunos com dificuldades de aprendizagem em tempos de ensino remoto. Research. Soc Dev.

[23] Sodagar A, Javed R, Tahir H, Razak SIA, Shakir M, Naeem M (2022). Pathological features and neuroinflammatory mechanisms of SARS-CoV-2 in the brain and potential therapeutic approaches. Biomolecules.

[24] Lunardelo PP, Simões HO, Zanchetta S (2019). Diferenças e similaridades para P1 e N1. Rev CEFAC.

[25] Pelaquim A, Sanfins MD, Fornazieri MA (2023). Changes in auditory evoked potentials increase the chances of adults having central auditory processing disorder. Int Arch Otorhinolaryngol.

[26] Medeiros GM, Silva DPC, Pinheiro MMC (2020). Estudo do potencial evocado auditivo P300 antes e após o treinamento auditivo acusticamente controlado. Res Soc Dev.

