# Supplementary material for: Neurobiological activity of the auditory pathway and memory: a comparative study in individuals infected with SARS-Cov-2
Source: Codas. 2026 Mar 27;38(2):e20250056. doi: 10.1590/2317-1782/e20250056en (PMC13075828; doi:10.1590/2317-1782/e20250056en)
Supplement: Translation

# Translation: Atividade neurobiológica da via auditiva e memória: um estudo de comparação em indivíduos com infecção por SARS-Cov-2

**DOI:** 10.1590/2317-1782/e20250056pt

**Authors:** Liliane Razador Kerkhoff, Hélinton Goulart Moreira, Larissa Coradini, Fabiana Cristina Toillier, Cristiane Laís Piccoloto, Denis Altieri de Oliveira Moraes, Michele Vargas Garcia

**Affiliations:** 1 Programa de Pós-graduação em Distúrbios da Comunicação Humana, Departamento de Fonoaudiologia, Universidade Federal de Santa Maria – UFSM - Santa Maria (RS), Brasil.; 2 Curso de Fonoaudiologia, Universidade Federal de Santa Maria – UFSM - Santa Maria (RS), Brasil.

**Keywords:** SARS-Cov-2, Cognição, Adultos, Eletrofisiologia, Memória

## Abstract

**Objetivo:**

Analisar a influência do SARS-CoV-2 na memória e nos componentes do Potencial Evocado Auditivo de Longa Latência.

**Método:**

Estudo descritivo e quantitativo, aprovado pelo Comitê de Ética em Pesquisa, sob o número 56038322100005346. Para a composição amostral foram convidados 32 participantes com idades entre 18 e 35 anos, escolarizados, sem perda auditiva, integridade de orelha média, reflexos acústicos contralaterais presentes bilateralmente, sem alterações neurológicas ou psiquiátricas, com ou sem diagnóstico de SARS-CoV-2. Participaram do estudo 32 indivíduos, sendo 24 (75%) do sexo feminino e oito (25%) do sexo masculino, distribuidos em dois grupos. O Grupo Estudo (GE) foi composto por 15 indivíduos (53%) com diagnóstico de SARS-CoV-2 e o Grupo Controle (GC) por 17 participantes (47%) sem tal diagnóstico. Foram submetidos a anamnese, meatoscopia, avaliação audiológica básica, Potencial Evocado Auditivo de Longa Latência (PEALL) e ao Instrumento de Avaliação Neuropsicológica Breve-NEUPSILIN, utilizando o escore total para cada subtipo de memória. Para a análise estatística dos dados, foi utilizado o teste T-Student adotando p-valor <0,05.

**Resultados:**

Observou-se diferenças significantes com desempenho inferior para o grupo estudo em Memória Visual Episódico-Semântica (p-valor=0,014) e para Memória Visual de Curto Prazo (p-valor=0,042). No PEALL, houve diferença significativa apenas para o componente P1, com menor latência no GE.

**Conclusão:**

A infecção por SARS-CoV-2 influenciou negativamente na memória de adultos jovens, principalmente, na memória verbal episódico-semântica e memória visual de curto prazo. Ainda, o PEALL-verbal contribuiu no diagnóstico destes participantes demonstrando os efeitos deletérios da infecção.

## INTRODUÇÃO

A partir de dezembro de 2019, o mundo passou a viver uma situação preocupante no que tange a saúde da população, devido ao surgimento de um vírus com alta taxa de transmissão, que levou ao crescimento de inúmeros casos em todo o mundo. Em fevereiro de 2020, a Organização Mundial de Saúde (OMS)^(1)^ nomeou oficialmente a doença como COVID-19 (*Coronavírus Disease 2019*), bem como demonstrou as diversas consequências de tal infecção respiratória para a saúde global humana e em março do mesmo ano declarou pandemia.

Partindo da fisiopatologia da infecção viral e das diversas manifestações desta, mesmo após a fase aguda, em 2021, a OMS nomeou as diversas sequelas do vírus SARS-CoV-2 como a Síndrome Pós-Covid-19, COVID longa ou Sequelas pós-agudas de COVID-19 que definem pessoas que ainda apresentam sintomas após a infecção pelo vírus^(2-4)^.

Diante disso, sabe-se dos diversos impactos que o SARS-CoV-2 pode causar, como problemas relacionados a acuidade auditiva, sintomas otológicos como tontura e zumbido, dificuldades de compreensão de fala e até prejuízos referentes a memória, sendo esta frequentemente relatada na clínica fonoaudiológica. Desse modo, estes impactos podem ser relacionados às mudanças estruturais que manifestam-se em diferentes capacidades funcionais dos indivíduos. Por meio do bulbo, o vírus SARS-CoV-2, através do neurotropismo, consegue infectar e disseminar o vírus para o Sistema Nervoso Central (SNC), infectando regiões corticais e hipotalâmicas^(5)^. Uma vez que, o hipotálamo se conecta com grande parte das regiões cerebrais, podem ser manifestados prejuízos na atenção, funções executivas e memória do indivíduo^(6)^. Ainda, a perda de memória pode ser explicada pelo termo *Brain-Fog*, que se refere a um conjunto de sintomas neurológicos relacionados à COVID-19 e que gera em muitos pacientes uma perda de memória a curto e longo prazo^(2)^.

Para avaliar o Sistema Nervoso Auditivo Central (SNAC) temos o Potencial Evocado Auditivo de Longa Latência (PEALL). Este avalia a função auditiva em termos de processo sináptico em nível cortical e cognitivo, investigando habilidades como atenção e memória. Os potenciais obtidos através do PEALL são captados de forma objetiva e não invasiva. Os componentes P1, N1 e P2 refletem a chegada do estímulo ao córtex, já o componente N2 é relacionado com as habilidades de percepção, discriminação, reconhecimento e classificação do estímulo sonoro. Por fim, o P3 (ou P300), possibilita compreender se o indivíduo apresentou foco na realização da atividade proposta, refletindo sobre habilidades de atenção, discriminação, integração e memória^(7)^.

Para avaliar a funcionalidade cognitiva, o teste neuropsicológico breve NEUPSILIN^(8)^ tem como objetivo avaliar múltiplas habilidades cognitivas.

Na literatura, ainda não há estudos que verificaram os achados do NEUPSILIN com o PEALL como método de avaliação mais eficaz para o diagnóstico das queixas de memória em pacientes pós contaminação por SARS-CoV-2. Um estudo^(9)^ utilizou a avaliação neuropsicológica breve-NEUPSILIN e o Potencial Cognitivo - P300 com estímulo de fala, pré e pós treinamento auditivo cognitivo, para verificar se a sua estratégia terapêutica foi eficaz no tratamento de adultos com queixas de compreensão de fala e de cognição após infecção por COVID-19.

Dessa forma, este estudo se justifica pela necessidade de identificar qual técnica de avaliação diagnóstica (comportamental e eletrofisiológica) é mais eficaz para detectar alterações em uma população de adultos jovens com queixas relacionadas ao pós-COVID.

Logo, o objetivo do presente estudo foi analisar a influência do SARS-CoV-2 nos diferentes subtipos de memória e no Potencial Evocado Auditivo de Longa Latência (PEALL-verbal) em adultos jovens.

## MÉTODO

### Desenho do estudo

Trata-se de um estudo de caráter descritivo e quantitativo, aprovado pelo Comitê de Ética em Pesquisa, sob o número 56038322100005346. O Termo de Consentimento Livre e Esclarecido (TCLE) foi assinado por todos os participantes do estudo, visando o esclarecimento sobre os riscos e benefícios da sua participação.

Foram elencados os seguintes critérios de elegibilidade: indivíduos de ambos os sexos, com idades entre 18 e 35 anos, escolarizados, falantes do português brasileiro, preferência manual direita, limiares auditivos dentro dos padrões de normalidade, integridade de orelha média e reflexos acústicos contralaterais presentes bilateralmente e com ou sem diagnóstico de COVID-19, comprovados por meio da apresentação do teste RT-PCR.

Foram excluídos indivíduos com comprometimento neurológico ou psiquiátrico evidentes ou diagnosticados, musicistas, indivíduos expostos ao ruído ou com queixas otológicas (tontura e zumbido).

### Participantes

A amostra foi realizada por conveniência, do qual foram atendidos 85 indivíduos no ambulatório de audiologia de uma clínica escola, no período de setembro de 2021 a março de 2022. Destes, foram excluídos 47 participantes, devido a 36 apresentarem a percepção de zumbido crônico (42,35%), nove por apresentarem perda auditiva (10,58%), cinco (5,88%) pela impossibilidade de finalização das avaliações e dois (2,35%) devido a alteração de orelha média. Desse modo, contabilizaram-se 32 indivíduos, sendo 24 sexo feminino e oito do masculino, pareados quanto a idade, sexo e escolaridade e divididos em dois grupos:

Grupo experimental (GE), composto por 15 indivíduos (53%), com idades entre 19 e 27 anos (média: 22,54), sendo 12 mulheres e cinco homens, com escolaridade mínima de 12 anos e máxima de 18 anos (média: 15,38 anos), tendo diagnóstico de infecção por Sars-Cov-2 (comprovados por meio da apresentação do teste RT-PCR);

Grupo Controle (GC), composto por 17 participantes (47%), sendo 12 do sexo feminino e três do sexo masculino, com idades entre 19 e 34 anos (média: 22,79), com escolaridade mínima de 14 anos e máxima de 17 anos (média: 15,38 anos) e sem diagnóstico de infecção por Sars-Cov-2 (comprovados por meio da apresentação do teste RT-PCR).

### Delineamento metodológico

Os procedimentos foram divididos em: procedimentos para composição amostral e procedimentos de pesquisa. Todos os procedimentos do GE foram realizados em média seis meses após a infecção.

### Procedimentos para composição amostral:

Anamnese semi-estruturada: Todos os indivíduos preencheram um questionário online com seus dados de identificação, perguntas a respeito dos aspectos auditivos, queixas audiológicas de compreensão de fala, aspectos cognitivos, bem como de saúde no geral.

Inspeção Visual do Meato Acústico Externo: Realizada com o otoscópio, modelo TK da marca Mikatos, objetivando averiguar as condições necessárias de realização do exame subsequente, bem como a possível necessidade de encaminhamento ao profissional otorrinolaringologista.

Audiometria Tonal Liminar (ATL): Realizada em uma cabina acusticamente tratada, com fones de ouvido TDH-39. Para a pesquisa dos limiares auditivos, utilizou-se o audiômetro AD229, da marca *Interacoustics.* Foram avaliadas as frequências de 250Hz a 8000Hz, por via aérea, levando em consideração a normalidade proposta pela OMS^(10)^, ou seja, com limiares tonais até 20 dBNA.

Logoaudiometria: Realizada no mesmo equipamento da ATL. A avaliação foi realizada em duas etapas, ambas pesquisadas à viva voz. Primeiramente, realizou-se o Limiar de Reconhecimento de Fala (LRF), inicialmente em uma intensidade de 30 dBNA acima da média tritonal do indivíduo. Utilizou-se a técnica descendente-ascendente, considerando como limiar onde o indivíduo acertasse 50% das quatro apresentações. Em seguida, foi aplicado o Índice Percentual de Reconhecimento de Fala (IPRF), fixando a intensidade em 40 dBNA acima da média tritonal, considerando o conforto do participante. Para esse, foram apresentadas 25 palavras ao participante, que deveria repeti-las, sendo cada erro equivalente a 4%. Os erros foram subtraídos do total de 100%.

Medidas de Imitância Acústica (MIA): Foram realizadas no equipamento AT235, da marca *Interacoustics* e fone tipo TDH-39. Para a análise das curvas timpanométricas, foi utilizado o critério de classificação de Jerger, Jerger e Mauldin^(11)^, enquanto os reflexos acústicos foram classificados com base nos critérios de Jerger e Jerger^(12)^.

### Procedimentos de pesquisa

Instrumento de Avaliação Neuropsicológica Breve (NEUPSILIN): Foi utilizado para avaliar a habilidade cognitiva de memória, sendo uma ferramenta clínica que permite descrever de forma compreensiva os principais componentes das habilidades cognitivas ao longo das diferentes faixas etárias. Pode ser aplicado em indivíduos de 12 a 90 anos (da adolescência à senescência), com no mínimo um ano de escolaridade. O teste é composto por 32 tarefas, que visam caracterizar um perfil cognitivo breve, a partir de oito funções cognitivas principais: orientação têmporo-espacial, atenção concentrada auditiva, percepção visual, memória, habilidades aritméticas, linguagem, praxias e funções executivas.

O protocolo de avaliação é composto pelo manual de aplicação, o livro de estímulos, juntamente com materiais específicos para cada tarefa. No decorrer do teste, o avaliador pode repetir as instruções de cada subteste conforme necessidade do indivíduo, exceto nas tarefas de atenção e memória. Visto que o foco do presente estudo foi analisar a habilidade de memória, utilizou-se apenas este subteste como dado. O instrumento foi aplicado individualmente, com um avaliador dedicado a cada participante, e teve uma duração aproximada de 40 minutos, em um ambiente silencioso. Os valores de referência utilizados foram propostos no estudo de Fonseca et al.^(8)^.

Na avaliação de memória, o instrumento avalia os seguintes tipos: memória de trabalho (ordenamento ascendente de dígitos e span auditivo de palavras em sentenças), memória verbal episódica (imediata, tardia e reconhecimento), memória semântica (longo prazo), memória visual (curto prazo) e memória prospectiva. Em cada uma das atividades o indivíduo foi instruído de acordo com o livro de instruções. De acordo com as normas gerais do protocolo, nos subtestes avaliados neste estudo, para todas as tarefas e itens foi aceita uma única autocorreção.

A pontuação final foi obtida por meio do escore completo, para a habilidade de memória total. Para os subtipos de memória, utilizaram-se os escores totais, por meio da soma de cada subtarefa dentro dos tipos de memórias avaliadas, sendo que, quanto maior a pontuação, melhor é o desempenho cognitivo do participante.

Potencial Auditivo de Longa Latência-verbal (PEALL): O PEALL-verbal foi aplicado para a comparação entre os dois grupos, com o intuito de obter informações sobre o funcionamento neural do SNAC, por meio da análise das amplitudes (em *microvolts*) e da relação das ondas P2/P1. O equipamento utilizado foi o Smart EP da *Intelligent Hearing System* e os eletrodos foram posicionados nos pontos Fpz, A1, A2 e Cz.

Foram apresentados 150 estímulos verbais (*sweeps*), em uma intensidade de 70 dBNA, compostos pela sílaba /ba/, que representou o estímulo frequente (80% das vezes), e pela sílaba /di/, que representou o estímulo raro (20% das vezes), no paradigma *oddball* tradicional. A velocidade do estímulo foi de 1.1/sec, com filtro de 1-30 Hz, ganho de 100K e janela de tempo de 510 ms. Os indivíduos mantiveram-se em estado de alerta e prestaram atenção nos estímulos "raros", bem como realizaram a contagem desses estímulos percebidos mentalmente.

As orelhas foram pesquisadas de modo binaural desencadeando respostas da orelha direita e esquerda. A partir da estimulação, foram geradas duas ondas (onda frequente e onda rara) e realizada a análise da latência e amplitude dos potenciais em cada orelha posteriores comparações. Os potenciais corticais (P1, N1, P2) e o potencial misto (N2) foram marcados no traçado frequente e o potencial cognitivo (P300) marcado no traçado raro. Os valores de referência utilizados foram propostos no estudo de Bruno et al.^(13)^ e Didoné et al.^(14)^.

### Análise estatística dos dados

De acordo com o tamanho final dos grupos, foi computado através do programa G*Power, o poder obtido pelo teste para comparação de médias entre dois grupos independentes. Os parâmetros utilizados foram um tamanho de efeito grande (0.8), nível de significância de 5% e os tamanhos de amostras fixados em 15 e 17 indivíduos. Dessa forma, o poder estimado final do teste é de aproximadamente 71%, sendo considerado um nível satisfatório de precisão. Os dados foram tabulados em planilha eletrônica *Excel* e importados para análise estatística e quantitativa no programa SPSS v. 22^(15)^. A suposição de normalidade das variáveis foi verificada pelo teste de Kolmogorov-Smirnov, não tendo sido rejeitada esta hipótese para nenhuma variável do estudo (p > 0,05). Deste modo, foi utilizado o teste T para amostras independentes, considerando como valor de significância estatística p ≤ 0,05.

## RESULTADOS

Na Tabela 1, para observar a homogeneidade da amostra realizou as comparações referentes às variáveis entre os grupos, não demonstrando diferenças estatisticamente significantes.

**Tabela 1:** Análise das variáveis idade e escolaridade entre ambos os grupos

**VARIÁVEIS**	**GRUPO**	**N**	**MIN**	**MÁX**	**MÉDIA**	**DP**	**P-VALOR**
IDADE	GC	17	19,0	34,0	22,5	3,9	0,997
	GE	15	19,0	27,0	22,5	2,2	
ESCOLARIDADE	GC	17	14,0	17,0	14,9	1,1	0,213
	GE	15	12,0	18,0	15,5	1,5	

**Legenda:** GC = Grupo Controle; GE = Grupo experimental; MIN = mínimo; MÁX = máximo; DP = desvio padrão; N = número de participantes.

Na Tabela 2, foram realizadas as comparações entre o escore total e subtipos de memórias, evidenciando diferenças significantes entre a memória verbal episódico-semântica e visual de curto prazo, com menores escores no grupo com infecção por SARS-CoV-2.

**Tabela 2:** Análise dos diferentes subtestes de Memória no Neupsilin

**VARIÁVEIS**	**GRUPO**	**N**	**MIN**	**MÁX**	**MÉDIA**	**DP**	**P-VALOR**
Memória de Trabalho	GC	17	20,0	36,0	28,6	4,1	0,341
	GE	15	21,0	36,0	30,1	4,4	
Memória Verbal Episódico-Semântica	GC	17	16,0	35,0	24,8	6,7	**0,063** ^*^
	GE	15	16,0	28,8	21,0	3,4	
Memória Semântica de Longo Prazo	GC	17	4,0	5,0	4,8	0,4	0,260
	GE	15	4,0	5,0	4,9	0,2	
Memória Visual de Curto Prazo	GC	17	3,0	3,0	3,0	-	0,041*
	GE	15	2,0	3,0	2,8	0,4	
Memória Prospectiva	GC	17	1,0	2,0	1,9	0,4	0,487
	GE	15	1,0	2,0	1,9	0,2	
Memória Total	GC	17	45,0	79,0	63,1	10,0	0,374
	GE	15	47,0	72,0	60,4	6,9	

* Diferença significante

**Legenda:** GC = Grupo Controle; GE = Grupo experimental; MIN = mínimo; MÁX = máximo; DP = desvio padrão; N = número de indivíduos

Na Tabela 3, foi realizada a comparação das médias entre os grupos para os componentes do PEALL-verbal, demonstrando haver diferença significativa entre os grupos para latência e amplitude do componente P1. Destaca-se a diferença entre o número de orelhas, devido a alguns participantes de pesquisa apresentarem ausência do potencial. Mesmo que a estimulação tenha sido binaural, obteve-se respostas das duas orelhas, no entanto, observa-se na tabela que as orelhas direita e esquerda foram aglutinadas, pois não houve diferença significativa entre elas na comparação da latência e amplitude dos componentes entre as orelhas.

**Tabela 3:** Análise do PEALL-verbal entre os grupos

**VARIÁVEIS**	**GRUPO**	**N**	**MIN**	**MÁX**	**MÉDIA**	**DP**	**P-VALOR**
P1 - L	GC	33	50,0	95,0	71,6	12,6	0,034*
	GE	29	36,0	110,0	59,9	16,7	
P1 - A	GC	33	0,5	5,8	2,9	1,6	**0,004** ^*^
	GE	29	1,1	6,9	4,5	1,3	
N1 - L	GC	34	90,0	148,0	110,5	10,6	0,745
	GE	30	86,0	149,0	108,9	15,8	
N1 - A	GC	34	2,1	8,5	5,3	2,0	0,290
	GE	30	2,8	11,5	6,2	2,2	
P2 - L	GC	34	155,0	202,0	179,2	12,7	0,374
	GE	30	149,0	224,0	174,1	18,2	
P2 - A	GC	34	0,9	13,2	6,0	3,4	0,396
	GE	30	1,0	10,0	5,2	2,5	
N2 - L	GC	33	217,0	318,0	255,0	24,2	0,504
	GE	30	205,0	296,0	248,8	25,8	
N2 - A	GC	33	0,2	8,7	3,5	2,9	0,991
	GE	30	0,4	9,1	3,5	2,4	
P3 - L	GC	34	226,0	359,0	297,9	37,6	0,134
	GE	24	226,0	384,0	318,8	36,5	
P3 - A	GC	34	1,9	10,8	5,6	2,7	0,625
	GE	24	1,1	16,0	5,0	4,0	

* Diferença significante

**Legenda:** L = latência; A = Amplitude; GC = Grupo Controle; GE = Grupo experimental; MIN = mínimo; MÁX = máximo; DP = desvio padrão; N = número de orelhas

Na figura 1, é possível verificar a representação do PEALL-verbal para ambos os grupos.

**Figura 1:** Representação do PEALL-verbal para ambos os grupos

Fonte: Imagem do acervo pessoal do pesquisador

Na tabela 4, foi realizada a análise da presença e ausência dos componentes por grupo e soma total das orelhas, demonstrando uma ocorrência maior de ausências para o P3 em relação ao GE.

**Tabela 4:** Análise de frequência relativa das ocorrências de presenças e ausências dos componentes do PEALL entre os grupos

**PEALL-(ms)**	**Ocorrência**	**GE (30 orelhas) N %**	**GC (34 orelhas) N %**
**P1**	Ausência	1 3,34%	1 2,94%
	Presença	29 96,66%	33 97,06%
**N1**	Ausência	0 0%	0 0%
	Presença	30 100%	34 100%
**P2**	Ausência	0 0%	0 0%
	Presença	30 100%	34 100%
**N2**	Ausência	0 0%	1 2,94%
	Presença	30 100%	33 97,06%
**P3**	Ausência	6 20%	0 0%
	Presença	24 80%	34 100%

**Legenda:** GC: Grupo Controle; GE: Grupo experimental.

## DISCUSSÃO

A relevância deste estudo refere-se ao fato de inúmeros indivíduos no pós-infecção por SARS-CoV-2 apresentarem queixas de memória, sendo um sintoma persistente em 70% dos participantes analisados^(16)^. Assim, após avaliar tal queixa detalhadamente, os dados observados vão ao encontro da hipótese deste estudo de que, os indivíduos que apresentaram diagnóstico de SARS-CoV-2 possuem maior número de alterações de memória.

Pode ser observado na Tabela 1 que os participantes deste estudo são todos jovens e de alta escolaridade, o que torna ainda mais relevante avaliar a memória e o funcionamento neuronal do Sistema Nervoso Central (SNC) no pós infecção por SARS-CoV-2. Nesse contexto, entender o funcionamento do SNC nessa população pode ajudar a identificar possíveis discrepâncias no processamento de informações ou sinais precoces de alterações neuropsicológicas, contribuindo para a prevenção de distúrbios cognitivos ao longo da vida.

Na Tabela 2, observam-se piores achados nos diferentes subtipos de memória para o grupo no pós infecção por SARS-CoV-2, com duas das seis tarefas apresentando significância estatística, sendo elas a memória verbal episódica-semântica e a memória visual de curto prazo. Este dado concorda com estudiosos que demonstraram defasagem na habilidade de memória verbal episódica em indivíduos pós infecção por SARS-CoV-2, quando comparados a pacientes saudáveis ou assintomáticos^(17-19)^. Ainda, os dados aqui expostos estão em consonância com o estudo de Bhattacharyya et al.^(20)^, que referiram o quanto o indivíduo no pós infecção por SARS-CoV-2 poderia apresentar redução do estado de alerta.

Outro estudo realizado cinco meses após alta hospitalar por SARS-CoV-2 demonstrou anormalidades cognitivas nos indivíduos, sendo que cerca de 20% da amostra apresentou disfunções de memória verbal após tal infecção^(21)^. Isto ocorre devido aos processos verbais exigirem memória, pois sua função básica é estruturar o pensamento de maneira ordenada, permitindo a retenção, recordação e reprodução dos conteúdos^(22)^.

No que diz respeito à memória visual de curto prazo, há relação da fisiologia da memória explícita, a qual, é dependente de mecanismos como: hipocampo, amígdala cerebral e córtex pré frontal, principalmente. Estudos relatam que há danos cerebrais causados pelo SARS-CoV-2, destacando processos inflamatórios e morte de neurônios^(23)^, justificando mais uma vez os achados da Tabela 2.

Sobre os dados do PEALL-verbal observados na Tabela 3, com diferença entre os grupos no Potencial P1, podemos observar que, obteve-se menor latência e maior amplitude para o GE. Este dado nos demonstra uma maior velocidade em ativação neural (ms) e maior recrutamento neural (amplitude) em córtex auditivo primário. Aqui observamos que o aumento de amplitude em relação ao grupo GE pode não ser um fator positivo. O estudo de Oliveira et al*.* ^(7)^ encontrou maiores amplitudes para P1 em idosos em comparação ao grupo de adultos, demonstrando maior robustez de resposta neural, para compensar outros possíveis déficits. Eles sugeriram que uma amplitude maior do potencial cortical traduz um maior esforço auditivo que consequentemente ocasiona um maior cansaço levando ao esgotamento mental, o que pode estar relacionado ao comprometimento cognitivo, fazendo relação com as queixas de atenção e memória dos indivíduos estudados.

Ademais, sabe-se que o componente P1 é um biomarcador que pode ser identificado em todas as faixas etárias, e está diretamente relacionada com a detecção do som no córtex auditivo primário, sendo o primeiro registro do processamento do sinal sonoro^(24)^. Apesar do objetivo deste estudo, não ser sobre a avaliação do Processamento Auditivo Central, é conhecida e evidente a indissociabilidade das habilidades auditivas e cognitivas. Em outro estudo^(25)^, o P1 apresentou 57,5% de acurácia em relação à probabilidade de alteração no processamento auditivo, enquanto o P3, 55%. Os dados aqui expostos ainda concordam com o estudo acima, quanto a importância de analisar todos os componentes do PEALL e não apenas o P300.

Referente a análise do P300, traz informações referentes atenção, discriminação, integração e memória, assim demonstra sobre o uso funcional que indivíduo faz do estímulo auditivo. Assim, está altamente relacionado a habilidades cognitivas, principalmente atenção e a discriminação auditiva^(14)^. Frente a isso, no contexto do presente estudo, justificam-se os achados obtidos no potencial P3 apresentados na Tabela 4.

Ainda na Tabela 4, é possível observar que houve 20% de ausência do P300 no GE. Para garantir que o resultado estava fidedigno, o avaliador ao final questionava o paciente sobre o número de estímulos escutados. Tal achado corrobora com a queixa apresentada pelos participantes do grupo, ressaltando a importância da avaliação do P300. Esse potencial é um instrumento eficaz no monitoramento das mudanças ocorridas no Sistema Auditivo Central (SAC) sendo o seu atraso na latência ou a sua ausência, um indicativo de distúrbio relacionado a atenção e cognição^(26)^.

As limitações deste estudo incluem não ter sido realizada a avaliação comportamental do Processamento Auditivo Central, para caso tivessem alterações e interesse em realizar reabilitação, encaminhados para treinamento auditivo. Ainda não foi realizada uma análise de correlação entre esses achados comportamentais e eletrofisiológicos por indivíduos, como realizado no estudo de Oliveira et al.^(7)^.

Assim, esta pesquisa enfatiza a importância de incluir testes comportamentais e exames eletrofisiológicos no diagnóstico, uma vez que o NEUPSILIN e o PEALL-verbal auxiliaram nessa avaliação, permitindo uma intervenção mais precisa. Neste sentido, tais achados concordam com Soares et al.^(9)^, que apresentaram um modelo de treinamento auditivo cognitivo, com tarefas visuais, auditivas e com funções executivas para a população adulta após exposição a SARS-CoV-2, obtendo sucesso por meio de uma reabilitação integralizada (cognição + habilidades auditivas).

## CONCLUSÃO

A infecção por SARS-CoV-2 influenciou negativamente na memória de adultos jovens, principalmente, na memória verbal episódico-semântica e memória visual de curto prazo. Ainda, o PEALL-verbal contribui no diagnóstico destes indivíduos demonstrando os efeitos deletérios da infecção.
